# Supplementary material for: Cathepsin B aggravates atherosclerosis in ApoE-deficient mice by modulating vascular smooth muscle cell pyroptosis through NF-κB / NLRP3 signaling pathway
Source: PLoS One. 2024 Jan 2;19(1):e0294514. doi: 10.1371/journal.pone.0294514 (PMC10760722; doi:10.1371/journal.pone.0294514)
Supplement: S1 Raw images — (PDF) [file pone.0294514.s001.pdf]

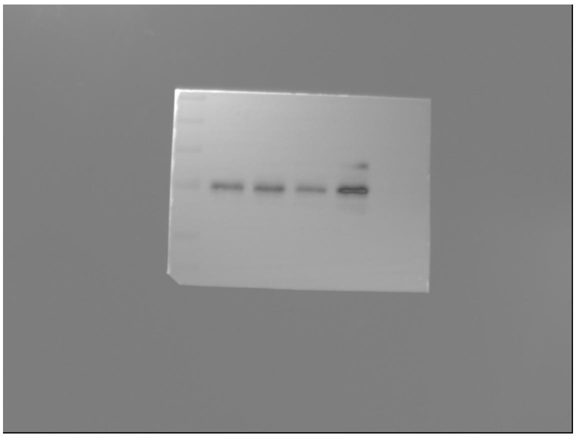

CTSB for Fig 1c

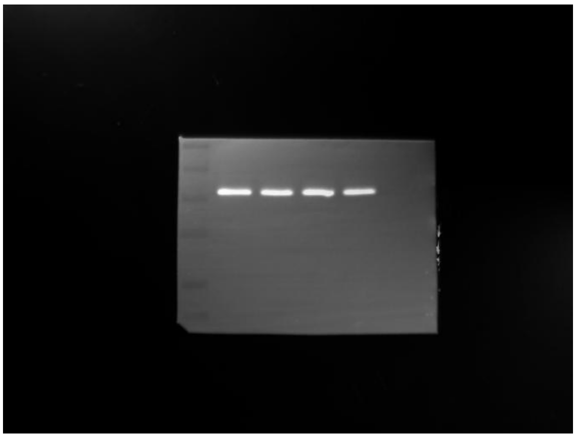

GAPDH for Fig 1c

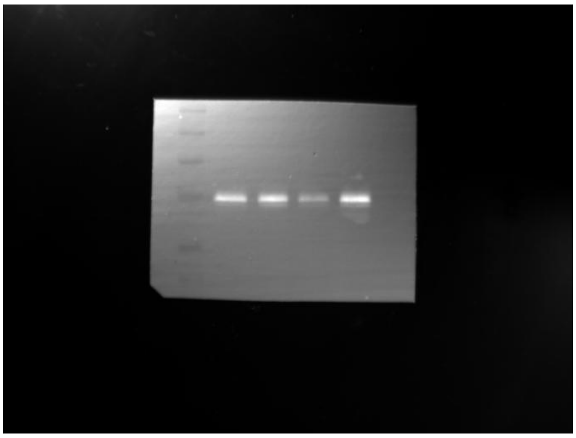

CTSB for Fig 1D

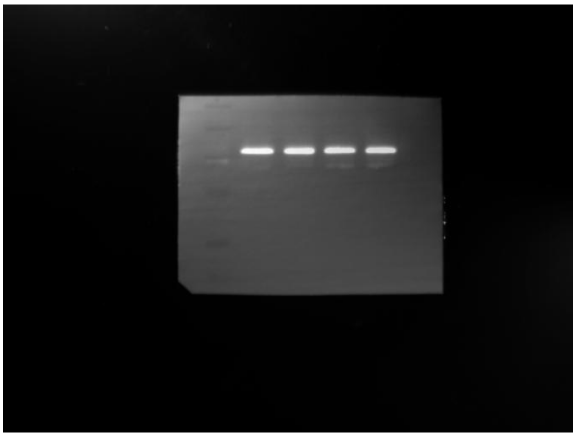

GAPDH for Fig 1D

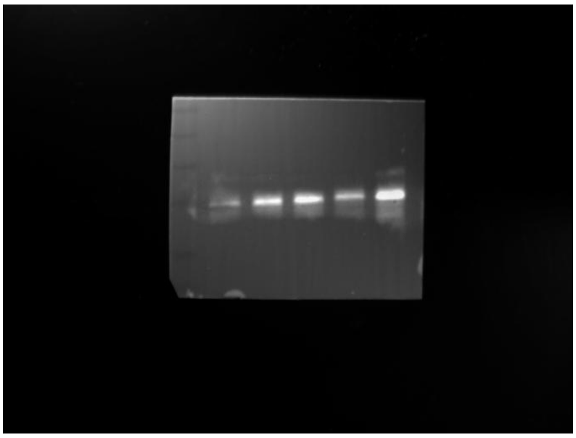

CTSB for Fig 2C

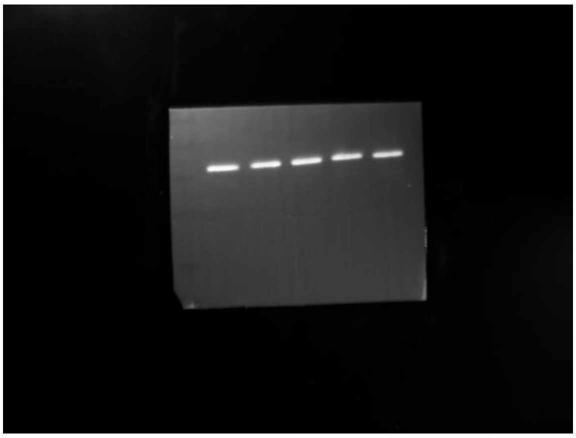

CTSB-GAPDH for Fig 2C

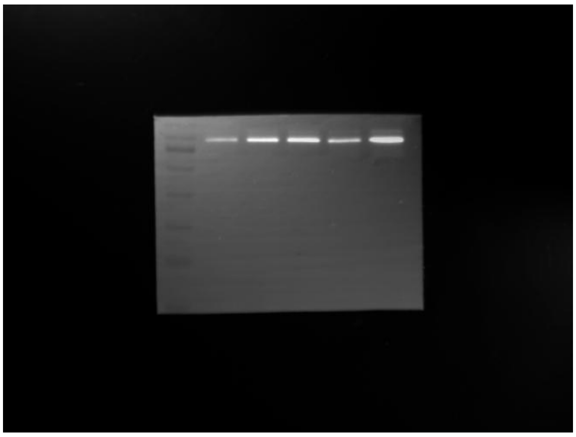

NLRP3 for Fig 2C

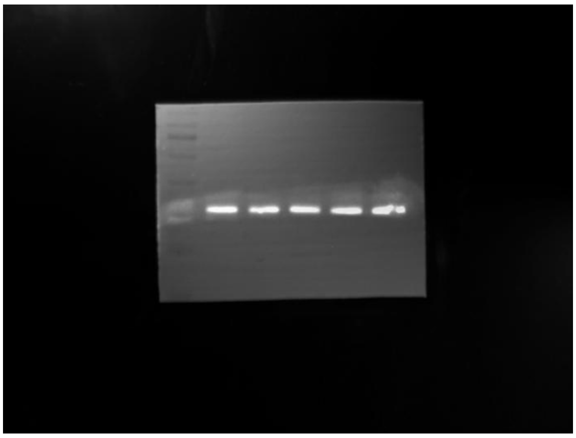

NLRP3-GAPDH for Fig 2C

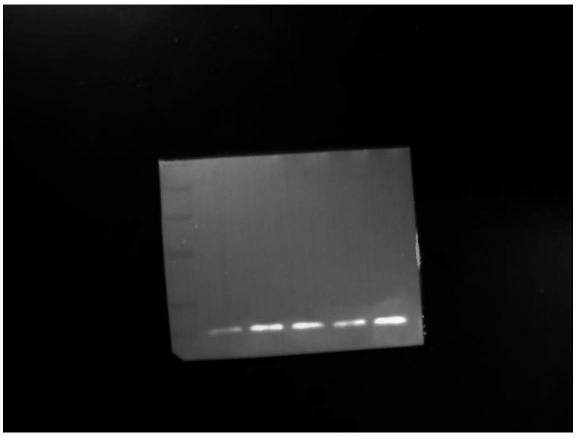

Caspase-1 p10 for Fig 2C

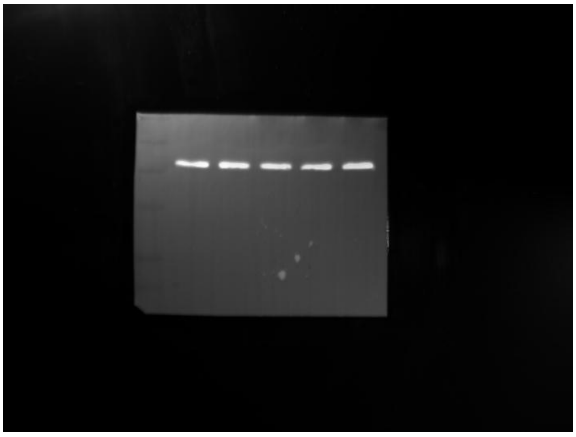

Caspase-1 p10-GAPDH for Fig 2C

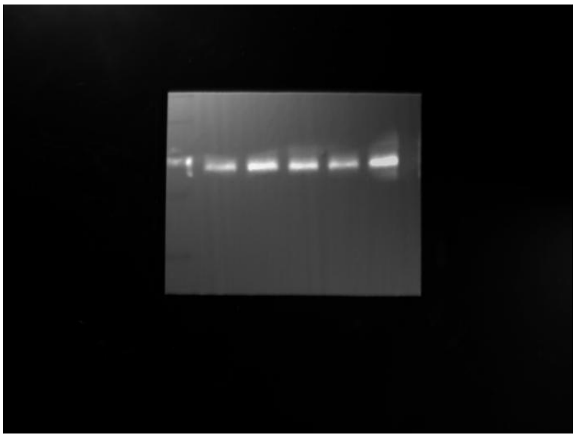

GSDMD-N for Fig 2C

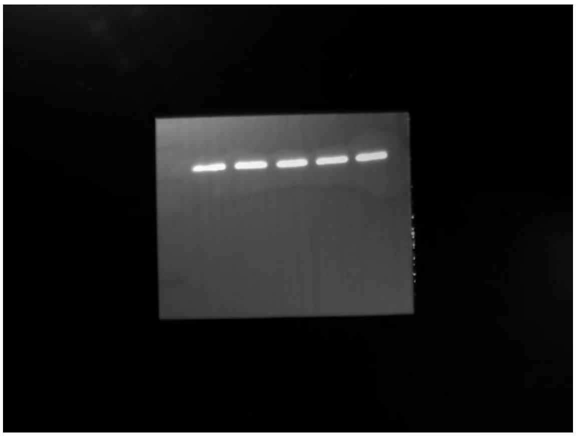

GSDMD-N-GAPDH for Fig 2C

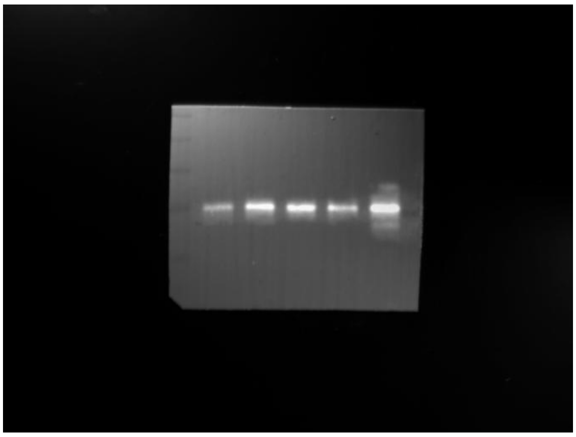

CTSB for Fig 4B

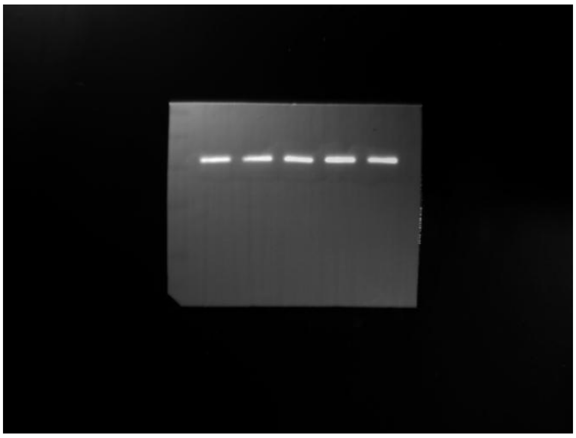

CTSB-GAPDH for Fig 4B

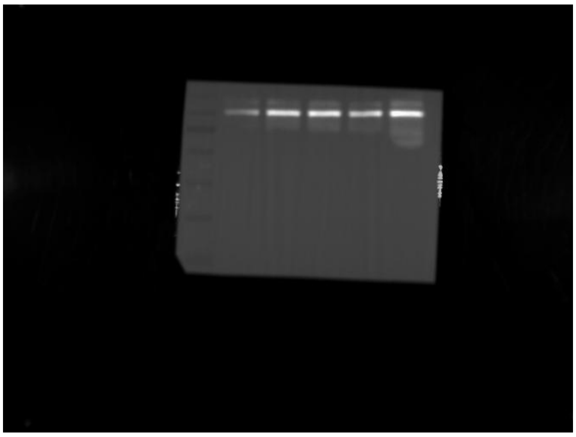

NLRP3 for Fig 4B

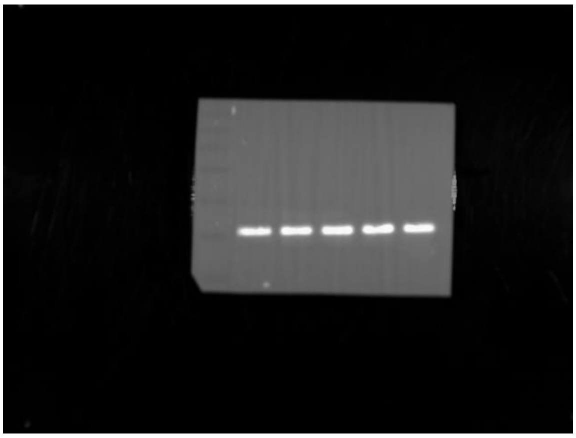

NLRP3-GAPDH for Fig 4B

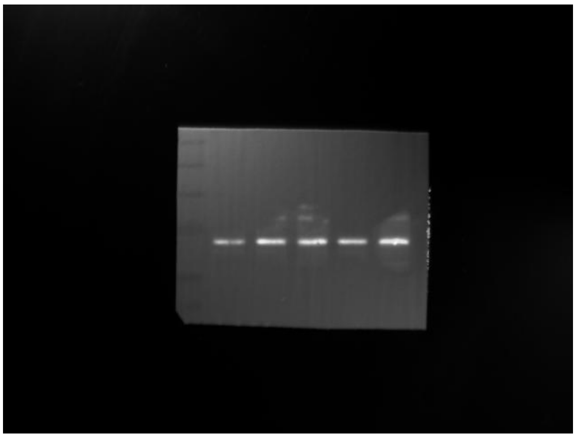

ASC for Fig 4B

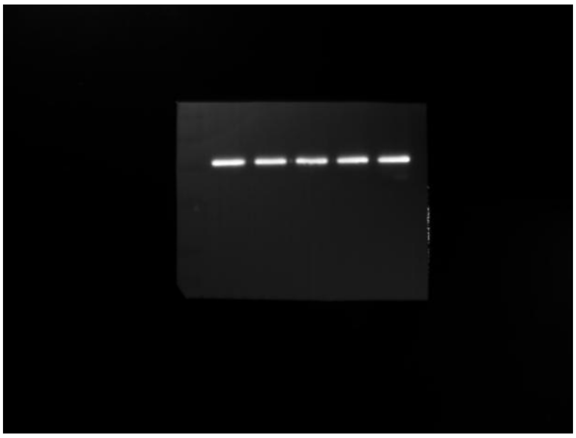

ASC-GAPDH for Fig 4B

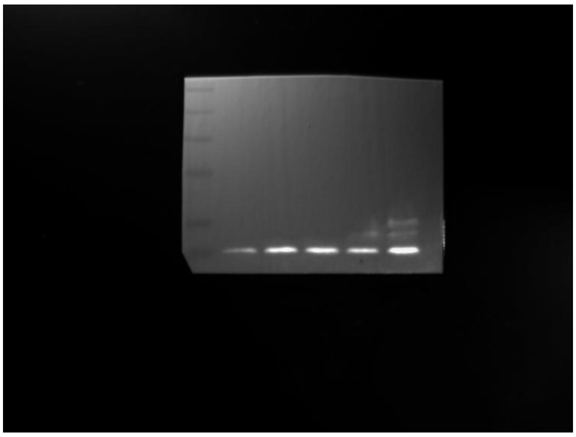

Caspase-1 p10 for Fig 4B

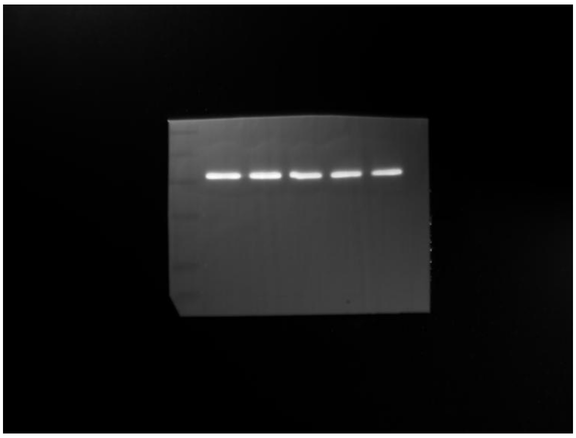

Caspase-1 p10-GAPDH for Fig 4B

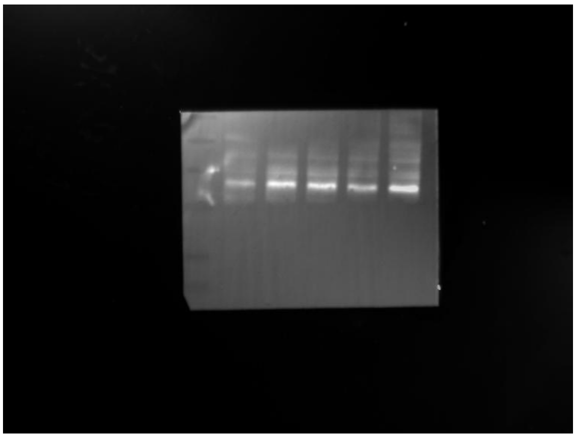

GSDMD-N for Fig 4B

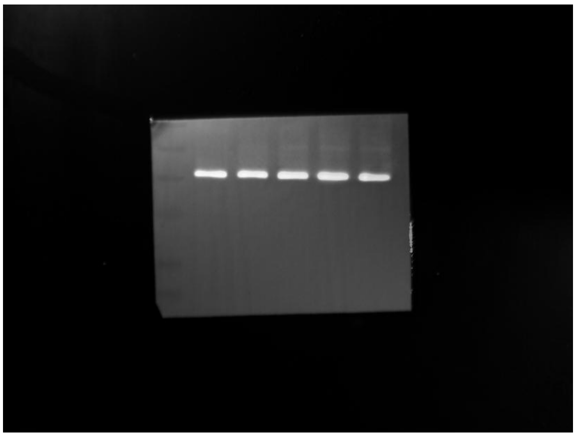

GSDMD-N GAPDH for Fig 4B

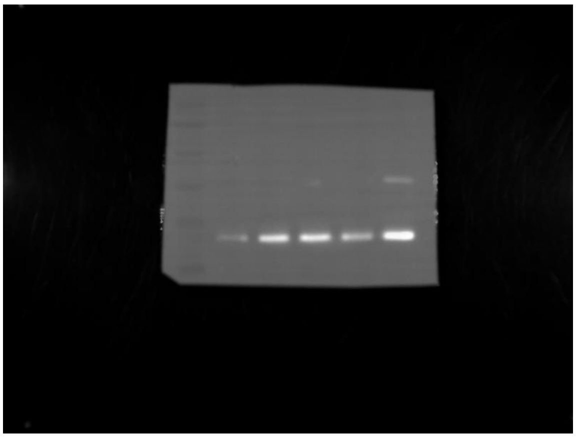

IL-18 for Fig 4B

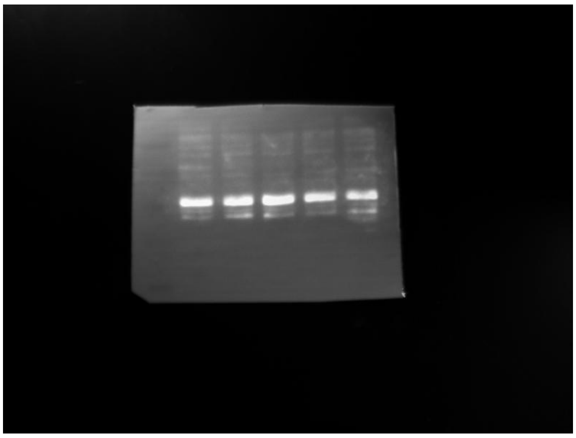

IL-18 GAPDH for Fig 4B

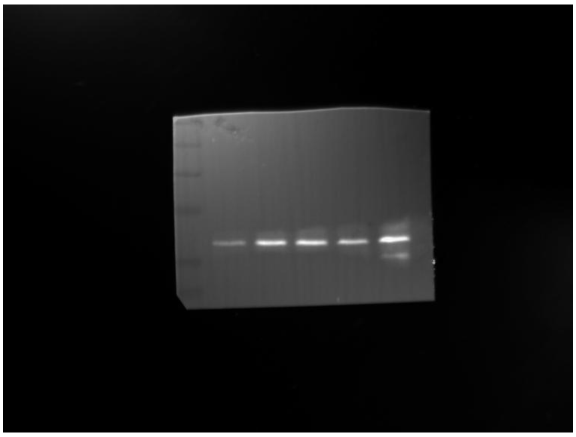

IL-1β for Fig 4B

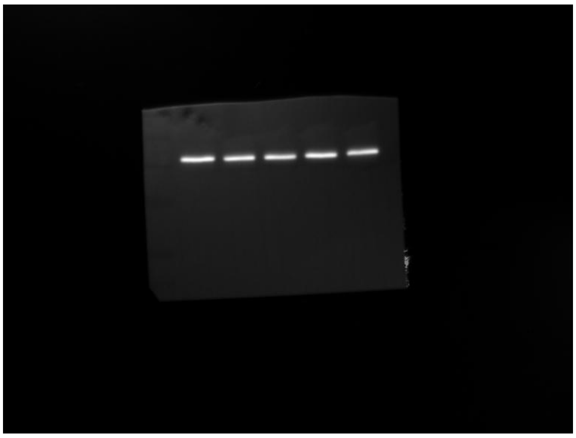

IL-1β GAPDH for Fig 4B

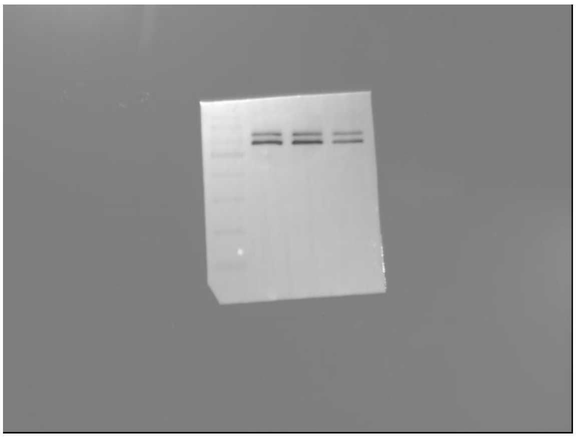

NLRP3 for Fig 6B

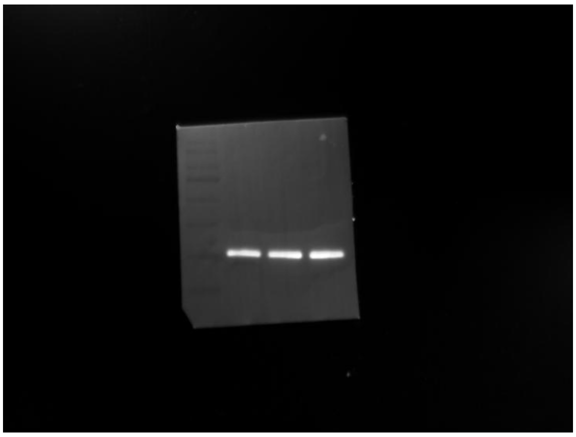

NLRP3 GAPDH for Fig 6B

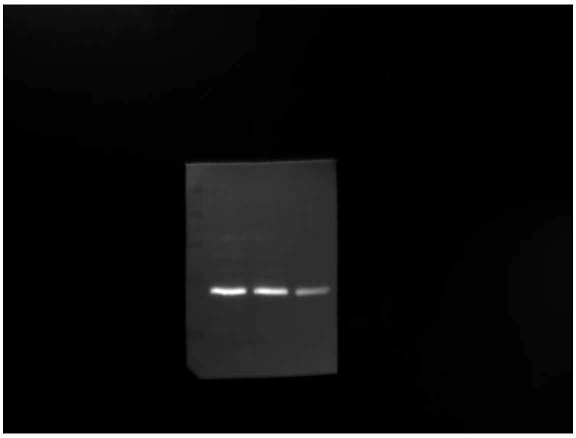

ASC for Fig 6B

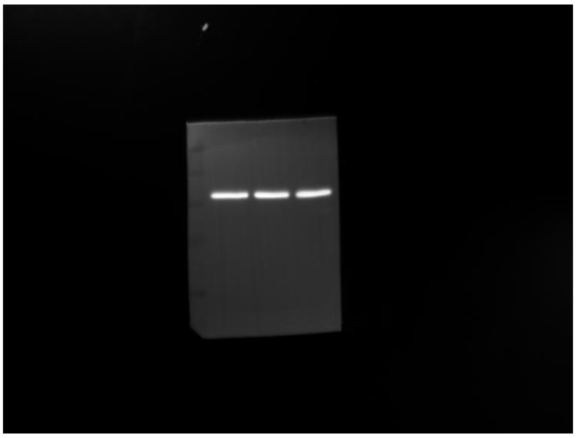

ASC GAPDH for Fig 6B

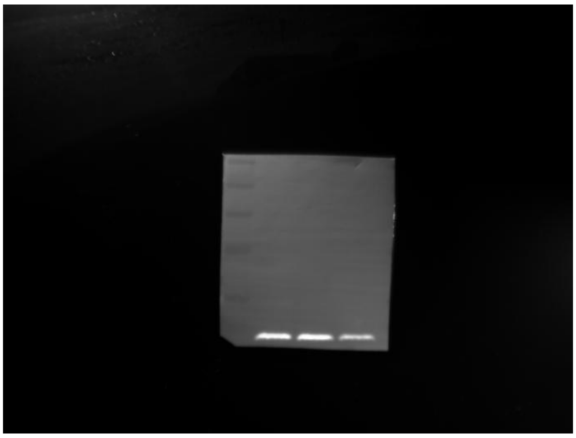

Caspase-1 p10 for Fig 6B

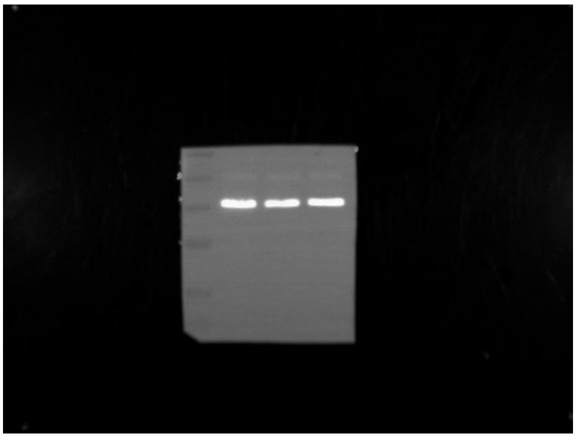

Caspase-1 p10 GAPDH for Fig 6B

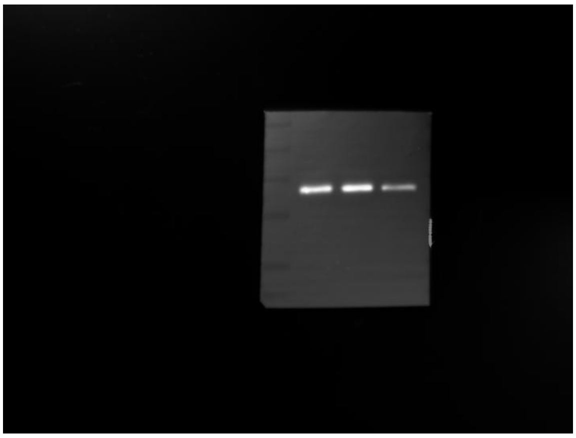

GSDMD-N for Fig 6B

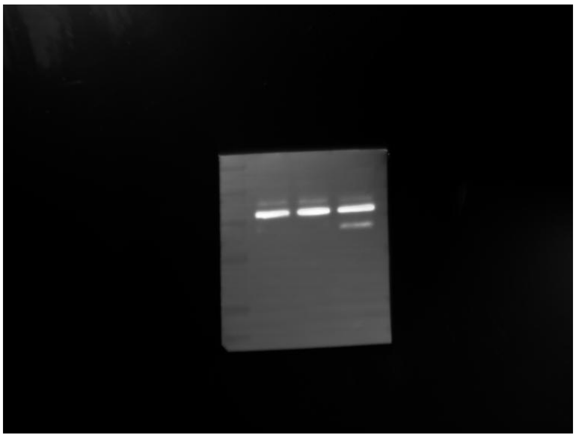

GSDMD-N GAPDH for Fig 6B

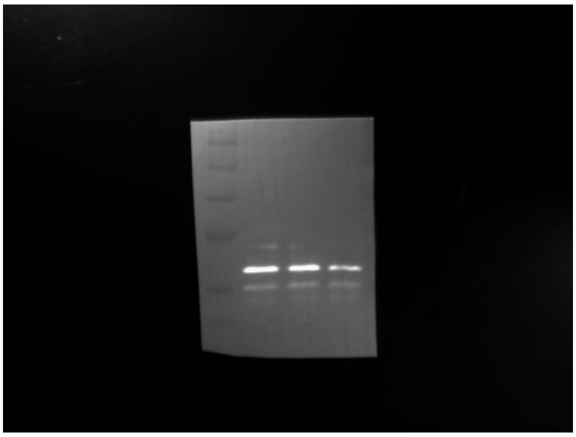

IL-1β for Fig 6B

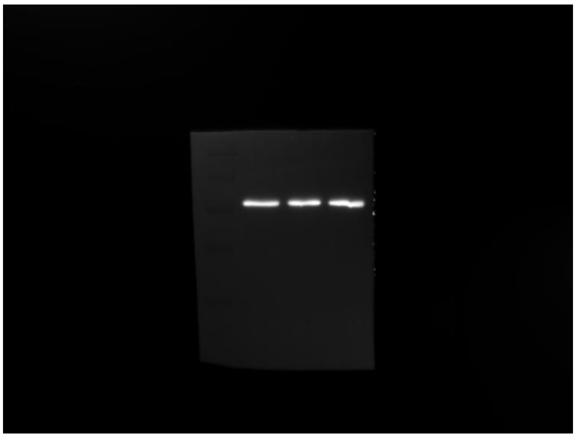

IL-1β GAPDH for Fig 6B

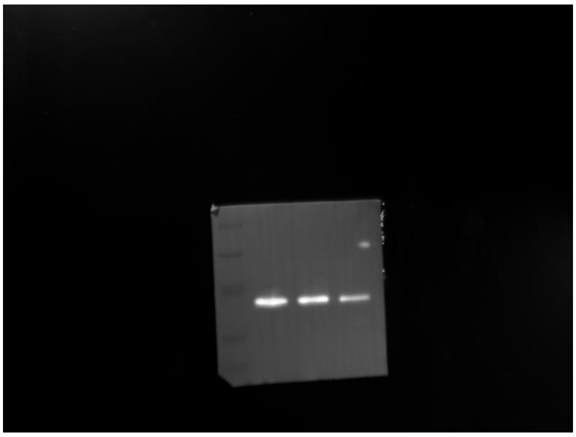

IL-18 for Fig 6B

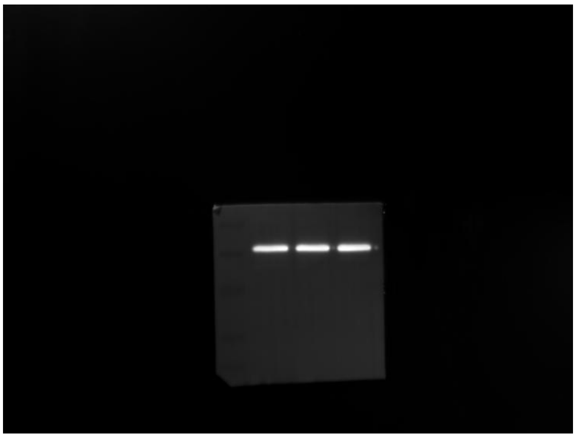

IL-18 GAPDH for Fig 6B

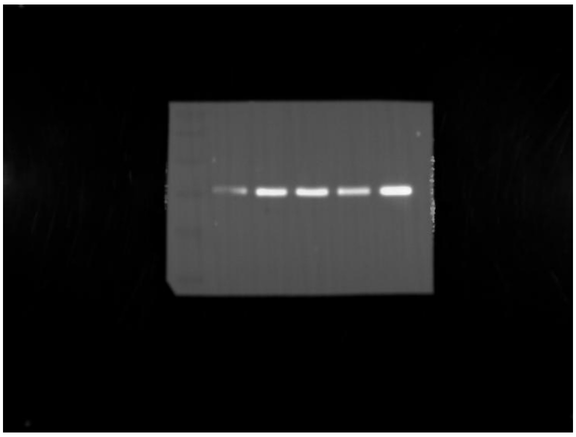

P-IKBα for Fig 6C

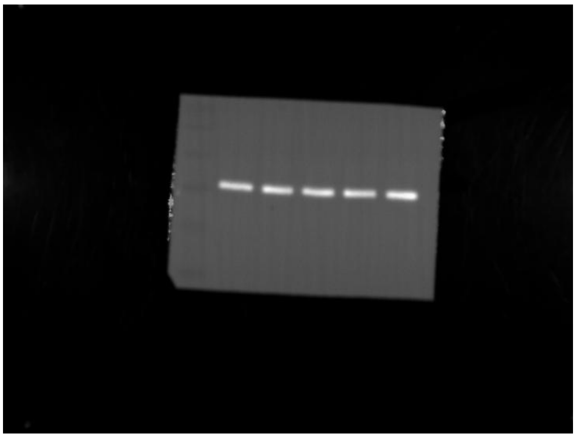

T-IKBα for Fig 5C

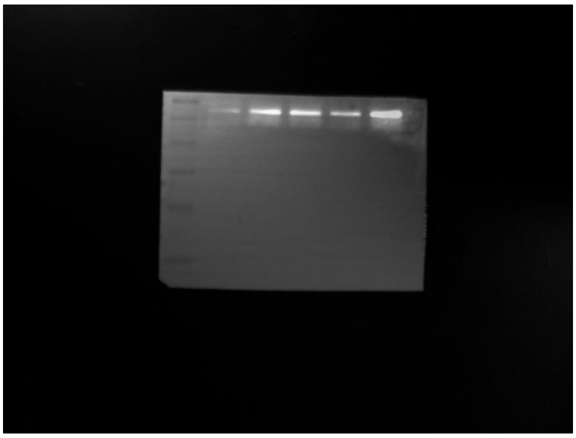

P-NFκBp65 for Fig 5C

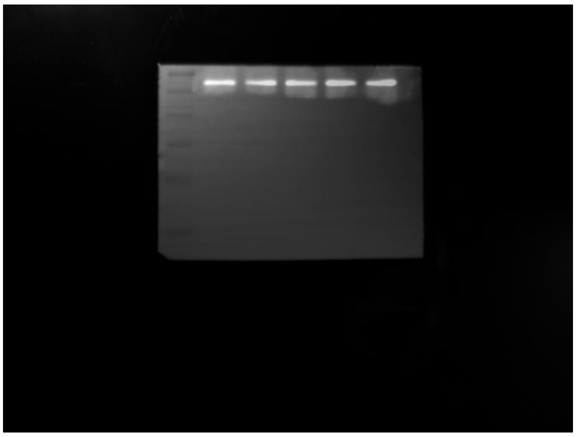

T-NFκBp65 for Fig 5C

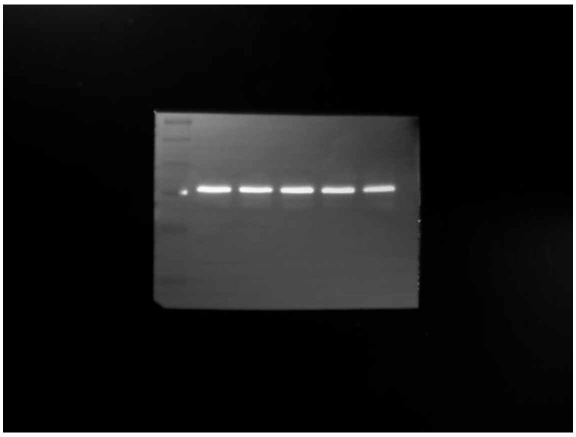

GAPDH for Fig 5C

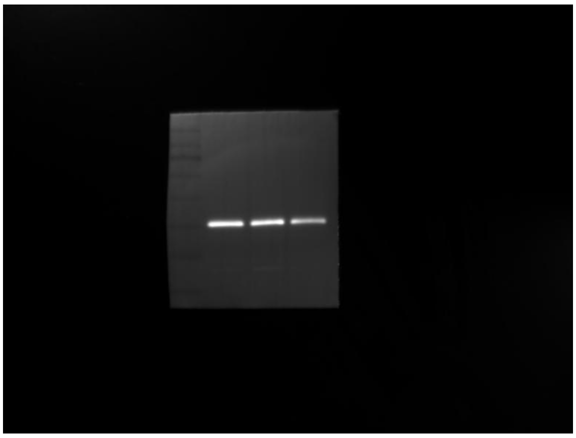

P-IKBα for Fig 5D

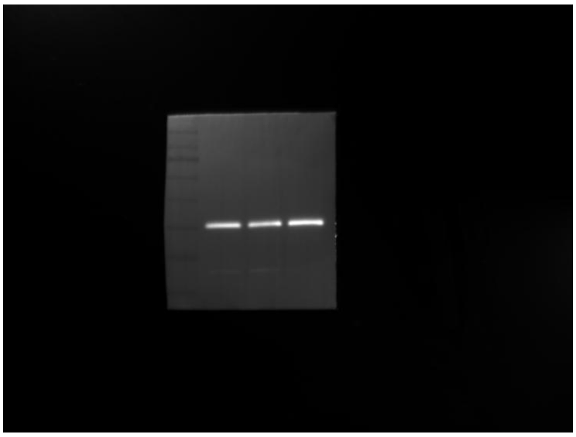

T-IKBα for Fig 5D

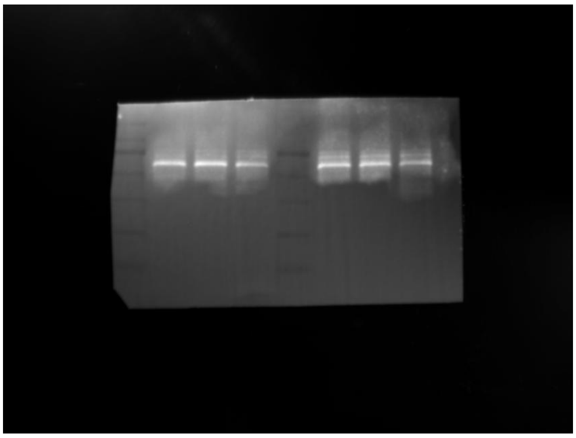

P-NFκBp65 for Fig 5D

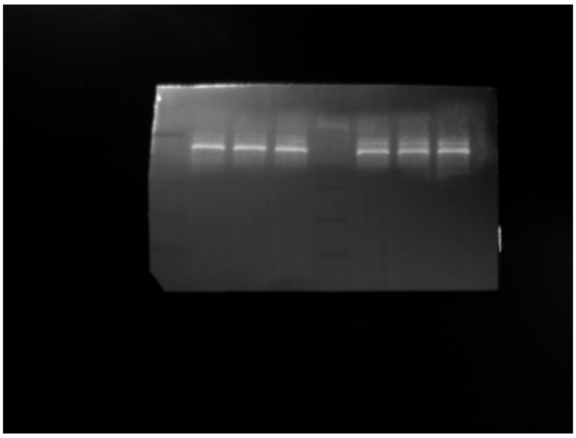

T-NFκBp65 for Fig 5D

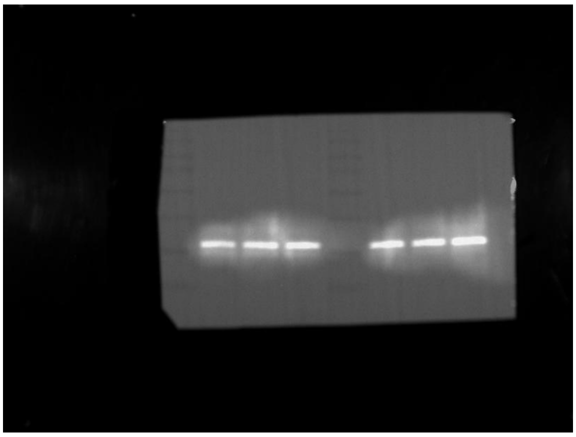

GAPDH for Fig 5D
