# Supplementary material for: Cathepsin B aggravates atherosclerosis in ApoE-deficient mice by modulating vascular smooth muscle cell pyroptosis through NF-κB / NLRP3 signaling pathway
Source: PLoS One. 2024 Jan 2;19(1):e0294514. doi: 10.1371/journal.pone.0294514 (PMC10760722; doi:10.1371/journal.pone.0294514)
Supplement: Attachment — Submitted filename: Response_to_Reviewers.docx [file pone.0294514.s002.docx]

August 29, 2023

Dear Professor Gou:

Re: Manuscript ID: PONE-D-23-17730

Title: Cathepsin B aggravates atherosclerosis in ApoE-deficient mice by modulating vascular smooth muscle cell pyroptosis through the NF-κB / NLRP3 signaling pathway.

Thank you for your letter and the reviewers' comments concerning our manuscript entitled “Cathepsin B aggravates atherosclerosis in ApoE-deficient mice by modulating vascular smooth muscle cell pyroptosis through the NF-κB / NLRP3 signaling pathway” (PONE-D-23-17730). Those comments are valuable and very helpful. We have read through them carefully and have made corrections. Based on the instructions provided in your letter, we uploaded the revised manuscript file. Revisions in the text are shown in red. In addition, the manuscript was reviewed, proofread, and edited by Editage, a professional language editing service. The responses to the academic editor and reviewer's comments are marked in blue and presented below.

Response to academic editor:

Journal Requirements:

When submitting your revision, we need you to address these additional requirements.

1. Please ensure that your manuscript meets PLOS ONE's style requirements, including those for file naming. The PLOS ONE style templates can be found at

https://journals.plos.org/plosone/s/file?id=wjVg/PLOSOne_formatting_sample_main_body.pdf and

https://journals.plos.org/plosone/s/file?id=ba62/PLOSOne_formatting_sample_title_authors_affiliations.pdf

Response: We have revised the manuscript according to the requirements of PLOS ONE style templates.

2. We suggest you thoroughly copyedit your manuscript for language usage, spelling, and grammar. If you do not know anyone who can help you do this, you may wish to consider employing a professional scientific editing service.

Response: We have comprehensively examined the language problems in the manuscript, and the manuscript was reviewed, proofread, and edited by Editage, a professional scientific editing service.

3. Please note that funding information should not appear in any section or other areas of your manuscript. We will only publish funding information present in the Funding Statement section of the online submission form. Please remove any funding-related text from the manuscript.

Response: We have deleted the funding information from the manuscript.

4.Please state what role the funders took in the study. Please include this amended Role of Funder statement in your cover letter; we will change the online submission form on your behalf.

Response: We have added the following information in the cover letter: "The funders had no role in the study design, data collection and analysis, decision to publish, or preparation of the manuscript."

5.In your Data Availability statement, you have not specified where the minimal data set underlying the results described in your manuscript can be found. "Upon re-submitting your revised manuscript, please upload your study’s minimal underlying data set as either Supporting Information files or to a stable, public repository and include the relevant URLs, DOIs, or accession numbers within your revised cover letter.

Response: Our study's minimal underlying data can be found in the Supporting Information files. This information was added to the revised manuscript.

6.We note that you have stated that you will provide repository information for your data at acceptance. Should your manuscript be accepted for publication, we will hold it until you provide the relevant accession numbers or DOIs necessary to access your data. If you wish to make changes to your Data Availability statement, please describe these changes in your cover letter and we will update your Data Availability statement to reflect the information you provide.

Response: We have provided the updated Data Availability statement in the cover letter and in the manuscript.

7.PLOS ONE now requires that authors provide the original uncropped and unadjusted images underlying all blot or gel results reported in a submission’s figures or Supporting Information files.

Response: Our study's original blot images can be found in the Supporting Information files which named “S1-raw-images”.

8. Please ensure that you refer to Figure 4 in your text as, if accepted, production will need this reference to link the reader to the figure.

Response: We apologize for our negligence in citing the figures incorrectly in the results section. We have rewritten sections 3.3 to 3.5 of the results, ensuring that all figures have been cited in the correct order.

*Response to Reviewers:

Reviewer #1:

Main points:

1. Several corrections are needed in the text including spelling errors (e.g., in Methods section: H&E and Masson STAINING, Immunohistochemistry STAINING), and lack of proper spacing between sentences in multiple places throughout.

Response: We are very sorry for these mistakes. We have made corrections according to the reviewer's comments and adjusted the text spacing appropriately.

2. In figure legends the following corrections are necessary-

- Fig 1 legend: E F G H are incorrectly referenced with respect to the order of the protein and mRNA data presented.

- Fig 2 legend: ‘G’ is LDL data (this is omitted); should be h-k, not g-j

Response: We apologize for not catching these errors when we proofread the manuscript. We have revised the Figure 1 and Figure 2 legends according to the reviewer's recommendations.

3. Fig 4 legend : remove the first sentence as not necessary.

Response: Indeed, as the reviewer indicated, the first sentence of Figure 4 is redundant, and we have deleted it.

4. Results section goes from Fig 4 to Fig 6 , with no mention of Fig 5. Need to include.

Response: We apologize for our negligence in citing the figures incorrectly in the results section. We have rewritten sections 3.3 to 3.5 of the results, ensuring that all figures have been cited in the correct order. Figure 5 has now been included.

5. Discussion: Should start with a clear statement of the findings of this study.

Response: Thank you for bringing this to our attention, we have rewritten the first paragraph of the discussion to state the findings of the study.

6. Final sentence in the Discussion should read “Targeted inhibition of CTSB-ACTIVATED Nf-kB…..

Response: We are very sorry for our error. We have made the necessary correction.

Reviewer #2:

Major Comments:

1) In the introduction of the paper, it is not clear why authors decided to study cathepsin. There are many other signaling molecules that are thought to be related to SMCs death, inflammatory responses, and atherosclerosis development. The authors need to add more rationale in the introduction to guide their readers. The authors need to first establish the potential link between cathepsin and AS before delving further into the mechanism of cathepsin contribution within the context of AS.

Response: We agree that we did not clarify why we decided to study cathepsin B. To rectify that deficiency, we have added a brief literature review of the relationship between cathepsin B and atherosclerosis in the introduction and established a potential link between cathepsin B and atherosclerosis.

2) Have the authors compared the contribution of apoptosis vs. pyroptosis in their study model?

Response: Apoptosis and pyroptosis were not compared in the model in this study. Although both are considered programmed cell death, their mechanisms and detection methods are not the same. According to the mechanism involved, the occurrence of pyroptosis was determined by measuring the expression of pyroptosis pathway proteins; that of LDH, IL-18, and IL-1β; and TUNEL / caspase-1 immunofluorescence co-staining.

3) What was the reason to only use male mice? Why didn’t the authors include female mice in their study? If the authors are interested in clinical relevance of their findings, the inclusion of female mice and the impact of female sex hormone in the inflammatory process of pyroptosis seems to be important.

Response: To our knowledge, sex hormones play an important role in the development of atherosclerosis. As the reviewer indicates, estrogen is important in the process of inflammation that promotes atherosclerosis. Some studies have shown that estrogen decreases adhesion molecule expression, neutrophil infiltration, and secretion of pro-inflammatory cytokines such as TNFα, IL-1β, and IL-6, thereby halting atherosclerosis in female mice [1,2]. Previous studies have shown that estrogen is atheroprotective in young women and in ovariectomized female mice and rats due to anti-inflammatory and vasoprotective effects [3–5]. However, in the most frequently used atherosclerotic mouse models, *Apolipoprotein E* knockout (*Apoe^−/−^*) and *low-density lipoprotein receptor* knockout (*Ldlr^−/−^*) mice, females up to 6 months old fed normal chow or atherosclerotic diet generally have a greater atherosclerotic lesion size and overall burden compared with their male counterparts. In 8+-month-old *Apoe*^−/−^ mice on normal chow or atherosclerotic diet, males were reported to have equal or larger plaques than females [6,7]. The animal model used in this study was the *Apoe^−/−^* mouse, which was studied until 20 weeks of age, which is less than 6 months. Considering that gender differences could potentially impact the experimental results, we decided to only utilize male mice at this time.Reviewer 's suggestion provides us with a new idea. Next time we will try the response of female mice to this study and compare the results of male and female mice to improve clinical relevance of their findings.

References for the above response:

1. Arenas IA, Armstrong SJ, Xu Y, Davidge ST. Chronic tumor necrosis factor-alpha inhibition enhances NO modulation of vascular function in estrogen-deficient rats. Hypertension. 2005 Jul;46(1):76-81. doi: 10.1161/01.HYP.0000168925.98963.ef. Epub 2005 May 23. PMID: 15911738.

2. Evans BR, Yerly A, van der Vorst EPC, Baumgartner I, Bernhard SM, Schindewolf M, Döring Y. Inflammatory Mediators in Atherosclerotic Vascular Remodeling. Front Cardiovasc Med. 2022 May 4;9:868934. doi: 10.3389/fcvm.2022.868934. PMID: 35600479; PMCID: PMC9114307.

3. Bakir S, Mori T, Durand J, Chen YF, Thompson JA, Oparil S. Estrogen-induced vasoprotection is estrogen receptor dependent: evidence from the balloon-injured rat carotid artery model. Circulation. 2000 May 23;101(20):2342-4. doi: 10.1161/01.cir.101.20.2342. PMID: 10821807.

4. Miller AP, Feng W, Xing D, Weathington NM, Blalock JE, Chen YF, Oparil S. Estrogen modulates inflammatory mediator expression and neutrophil chemotaxis in injured arteries. Circulation. 2004 Sep 21;110(12):1664-9. doi: 10.1161/01.CIR.0000142050.19488.C7. Epub 2004 Sep 7. PMID: 15353495.

5. Miller AP, Xing D, Feng W, Fintel M, Chen YF, Oparil S. Aged rats lose vasoprotective and anti-inflammatory actions of estrogen in injured arteries. Menopause. 2007 Mar-Apr;14(2):251-60. doi: 10.1097/01.gme.0000235366.39726.f6. PMID: 17194962.

6. Yerly A, van der Vorst EPC, Baumgartner I, Bernhard SM, Schindewolf M, Döring Y. Sex-specific and hormone-related differences in vascular remodelling in atherosclerosis. Eur J Clin Invest. 2023 Jan;53(1):e13885. doi: 10.1111/eci.13885. Epub 2022 Oct 22. PMID: 36219492.

7. Man JJ, Beckman JA, Jaffe IZ. Sex as a Biological Variable in Atherosclerosis. Circ Res. 2020 Apr 24;126(9):1297-1319. doi: 10.1161/CIRCRESAHA.120.315930. Epub 2020 Apr 23. PMID: 32324497; PMCID: PMC7185045.

4) The authors do not make the definition of the “Mock” group clear. Is the mock group infected with the GFP tagged neutral construct?

Response: We are very sorry for not clearly describing the “Mock” group. The mock group was transduced with the GFP-tagged construct that lacked the cathepsin B cDNA. Accordingly, we have defined the mock group in the Materials and Methods section 2.2.

5) The authors check the transfection efficiency 2 weeks after the construct delivery. Did the author also measure the construct expression in the mouse aorta at the end of 20 weeks and towards the end of experiments? Do they expect to see any drop in the expression level of those constructs?

Response: Thank you for the helpful comments. We also detected expression of the construct in mouse aortas at the 20th week. Due to space limitations, we only provided the western blot image of CTSB protein expression in Fig 2c, and the results showed that the expression of CTSB persisted at 20 weeks. We also detected EGFP in the aortas of the mice. The results showed that plaques appeared in the aortas of the virally transduced mice fed high-fat diet, and EGFP green fluorescence was present in the plaques. A representative image is shown below.

6) When measuring the efficiency of transfection, how the authors make sure that the detected green fluorescence is not coming from aortic wall elastin fibers. Elastin fibers are known to emit autofluorescence. How did the author make sure that the signal is specific to their constructs? Besides, the author did not include any image of non-transfected (NC) mice aorta as the negative control in figure 1A, so it is very hard to make sure that the green signal is due to transfection and not from non-specific elastin auto-fluorescence.

Response: Thank you for raising this point. Indeed, elastin fibers can emit autofluorescence. Thus, we have added a representative image of a non-transduced (NC) mouse aorta in Figure 1A and an image of non-transduced (NC) VSMCs in Figure 1B to increase the rigor of the results.

7) Figure 1A: when comparing the EGFP column for sh-CTSB vs. Lv-CTSB, there seems to be obvious differences in expression pattern. What could be the reason for this observation?

Response: We re-examined the original data of the Fig 1A sh-CTSB group and found that, as observed by the reviewer, the fluorescence in this group seemed to be less in the cytoplasm than in the elastin fibers. Thus, we examined more sections from this group and found better images where the intracellular EGFP fluorescence was higher. We speculate that the cause of this issue was related to fluorescence quenching. We replaced the image of the sh-CTSB group in Fig 1A, as shown below.

8) I have major concerns regarding all provided histological images. The authors are not clear on how the tissue sections were prepared (e.g., serial sections from the exact aortic area or random cross sections collected from different regions). They do not provide any clear description on how exactly these serial or non-serial sections were analyzed and if they had ROIs for each section. Also, there are no negative controls included in those image panels, so it is hard to determine how specific their staining is. Also, there is no quantification and actual statistical values and analysis provided for those immunohistochemistry or conventional histological staining provided throughout the figures.

Response: We are deeply sorry for our presentation of the histological images. The sections are serial sections from the aortic root area. All histological images were based on the aortic root with the aortic valve as a reference, and the plaque at the beginning of the valve was used as the ROI. We added magnified images to each group of HE- and Masson's trichrome-stained images. Plaque positive areas and ROIs are enclosed with black lines on the images. The positive Masson's staining area is indicated with black arrows. We are very sorry for our negligence in presenting these images. As you requested, we have also provided quantified data as mean ± SD with statistical comparisons.

9) The authors used cultured VSMCs within passages 5-15 for their experiments. Considering the high number of passages of SMCs after the initial seeding, did the author check those cells for alpha-actin SMC to ensure that the cultured cells still show the SMC phenotype?

Response: In this study, we originally did overlook the issue of SMC phenotype and did not detect the expression of α-SMA with respect to cell passage. Thus, we revived smooth muscle cells from the 5^th^ and 15^th^ passage and detected the expression of α-SMA by immunofluorescence. Statistical analysis showed that there was no significant difference in the expression of α-SMA between the 5^th^ and 15^th^ passage of the smooth muscle cells, suggesting that the SMC phenotype was maintained up to at least 15 passages. The results are as follows:

10) Figure 2: It is recommended to provide a magnified image of positively stained areas and use arrows to mark them.

Response: According to the reviewer's recommendation, we have provided magnified images of the positively stained areas and used arrows to identify them in Figure 2.

11) Figure 3A & 3B: aortic tissue cross sections presented in figure 3A and 3B do not correlate. Are these sections prepared from the same aortic sections?

Response: The sections in Figure 3A and 3B are not from the same aorta but are from the same treatment group.

12) In figure 3, the authors only provide images of IHC for markers of pyroptosis. However, they have not performed any image and statistical analysis to quantify the expression levels for those markers. Despite the lack of analysis, at the end of sub-section 3.4, the authors concluded that “CTSB aggravated pyroptosis of VSMCs in APOE-/- mice plaques”. I don’t believe that the provided evidence warrants such conclusions.

Response: We are very sorry for not providing image quantification and statistical analyses in Figure 3.We have now provided these data as indicated below.

13) I couldn’t find any reference in the results section to data presented in Figure 5.

Response: We apologize for our negligence in citing the figures incorrectly in the results section. We have rewritten sections 3.3 to 3.5 of the results, ensuring that all figures have been cited in the correct order. Figure 5 has now been included.

14) The authors claimed that SN50 treatment leads to decrease phosphorylation of IKB. To my knowledge, SN50 blocks the translocation and therefore activation of NFKBp65 into the nucleus. What is the relationship between SN50 mechanism of action and the pathway controlling IKB phosphorylation in the cytoplasm?

Response: To the best of our knowledge, SN50, as a synthetic polypeptide, prevents NF-κB nuclear translocation by competitively binding to the NFκB-p50 subunit in a concentration-dependent manner, and SN50 does not affect the degradation of IκB. Unfortunately, there is no report on SN50 affecting IκB phosphorylation. However, we know that the classical IκB phosphorylation process depends on IKK, and many inflammatory factors, including pyroptosis pathway factors such as IL-18 [1], IL-1β [2], and TNF-α [3], have been shown to promote IκB phosphorylation through IKK. In short, inflammatory factors / IKK / IκB / NF-κB can form a closed positive-feedback loop. On one hand, inflammatory factors promote NF-κB activation; on the other hand, activated NF-κB promotes the production of inflammatory factors. We can only speculate that SN50 blocks the positive-feedback loop formed by inflammatory factors / IKK / IκB / NF-κB by inhibiting NF-κB nuclear translocation and activation, thus indirectly affecting IκB phosphorylation. Wang et al. [4] found that SN50 reduced TNF-α-mediated p-p65, IKKα/β, p-IκB-α elevation in human umbilical vein endothelial cells. They also observed that SN50 reduced p-p65 and p-IκB-α, consistent with our study.

References for the above response are as follows:

1. Valente AJ, Yoshida T, Izadpanah R, Delafontaine P, Siebenlist U, Chandrasekar B. Interleukin-18 enhances IL-18R/Nox1 binding, and mediates TRAF3IP2-dependent smooth muscle cell migration. Inhibition by simvastatin. Cell Signal. 2013 Jun;25(6):1447-56. doi: 10.1016/j.cellsig.2013.03.007. Epub 2013 Mar 27. PMID: 23541442; PMCID: PMC3714795.

2. Cai J, Guan H, Jiao X, Yang J, Chen X, Zhang H, Zheng Y, Zhu Y, Liu Q, Zhang Z. NLRP3 inflammasome mediated pyroptosis is involved in cadmium exposure-induced neuroinflammation through the IL-1β/IkB-α-NF-κB-NLRP3 feedback loop in swine. Toxicology. 2021 Apr 15;453:152720. doi: 10.1016/j.tox.2021.152720. Epub 2021 Feb 13. PMID: 33592257.

3. Long J, Sun Y, Liu S, Yang S, Chen C, Zhang Z, Chu S, Yang Y, Pei G, Lin M, Yan Q, Yao J, Lin Y, Yi F, Meng L, Tan Y, Ai Q, Chen N. Targeting pyroptosis as a preventive and therapeutic approach for stroke. Cell Death Discov. 2023 May 10;9(1):155. doi: 10.1038/s41420-023-01440-y. PMID: 37165005; PMCID: PMC10172388.

4. Wang N, Jiang D, Zhou C, Han X. Alpha-solanine inhibits endothelial inflammation via nuclear factor kappa B signaling pathway. Adv Clin Exp Med. 2023 Feb 8. doi: 10.17219/acem/158781. Epub ahead of print. PMID: 36753375.

Minor Comments:

1) The manuscript needs some re-writing in proper scientific English language. Throughout the manuscripts there are so many errors with respect the proper English words or phrase and spacing in the beginning of sentences or before the brackets.

Response: We have comprehensively examined the language problems in the manuscript, and the manuscript was reviewed, proofread, and edited by Editage, a professional English language editing service.

2) When referring to other authors throughout the manuscript use the proper reference (e.g., Li et. al)

Response: We have made these corrections.

3) In the text, all “in vitro” or “in vivo” phrases should be in Italics.

Response: We have made these corrections.

4) Provide the full name of all markers and compounds for the first time in your text. Some of the abbreviated words are not known to all readers.

Response: Thank you for bringing this to our attention. We have made these corrections.

5) Throughout the manuscript there are many phrases that need to be corrected and revised. For example, “antigen repair” should be replaced with “antigen retrieval”, “cell-attached slides” with “tissue cross sections”, etc….

Response: As mentioned above, we have comprehensively examined the language problems in the manuscript, and the manuscript was reviewed, proofread, and edited by Editage, a professional English language editing service.

6) There seem to be redundancies in subsection 3.3 & 3.4 under the results section. I suggest the authors combine these two subsections.

Response: We have re-written these sections according to the reviewer's suggestion.

7) Figure 3: on the first line of legend, “plaue pyropsis” should be corrected to “plaque pyroptosis”.

Response: This has been corrected.

We have tried our best to improve the manuscript and make the recommended changes. These changes do not influence the content and framework of the paper but greatly improve the presentation of the data. We have marked the changes in red in the revised manuscript. We earnestly appreciate the editor's/reviewers' thoughtful suggestions and hope that the corrections will meet with your approval.

We thank you for allowing us to resubmit a revised copy of the manuscript and we highly appreciate your time and consideration. Please do not hesitate to contact us if there are any questions. Thanks again to the reviewers and editors for your hard work! Best wishes to you!

Sincerely.

Danan Liu.
