# Supplementary material for: Cathepsin B aggravates atherosclerosis in ApoE-deficient mice by modulating vascular smooth muscle cell pyroptosis through NF-κB / NLRP3 signaling pathway
Source: PLoS One. 2024 Jan 2;19(1):e0294514. doi: 10.1371/journal.pone.0294514 (PMC10760722; doi:10.1371/journal.pone.0294514)
Supplement: Attachment — Submitted filename: Response to Reviewers.docx [file pone.0294514.s003.docx]

October 12, 2023

Re: Manuscript ID: PONE-D-23-17730

Title: Cathepsin B aggravates atherosclerosis in ApoE-deficient mice by modulating vascular smooth muscle cell pyroptosis through NF-κB / NLRP3 signaling pathway

Dear Prof. Gou and Reviewers:

Thank you for your letter and the reviewers' comments concerning our manuscript (PONE-D-23-17730). Those comments are valuable and very helpful. We have read through the comments carefully and have made corrections. Based on the instructions provided in your letter, we uploaded the file of the revised manuscript. Revisions in the text are shown using red text. The manuscript was also proofread by Editage language services again. The responses to the Academic Editor's and Reviewer's comments are presented below, marked in blue text.

Response to Academic Editor :

Journal Requirements:

Please review your reference list to ensure that it is complete and correct. If you have cited papers that have been retracted, please include the rationale for doing so in the manuscript text, or remove these references and replace them with relevant current references. Any changes to the reference list should be mentioned in the rebuttal letter that accompanies your revised manuscript. If you need to cite a retracted article, indicate the article’s retracted status in the References list and also include a citation and full reference for the retraction notice.

Response: We have checked the list of references and have verified that it is correct and complete. Papers that have been retracted are not cited. This revision does not change the reference list.

*Response to Reviewers:

Reviewer #1:(No Response)

Reviewer #2:

Major Comments:

1) the authors still have not addressed the logic for not including the female group in their study. In their response to my major comment #3, they also further confirmed the fact that there are indeed differences between male and female mice in the extent of aortic plaque formation. But, they do not address their reasons for not including female groups in their study.

Response: We are sorry for the previous unclear description. Briefly, in the most frequently used atherosclerotic mouse models, *apolipoprotein E* knockout (*Apoe−/−*) and *low-density lipoprotein receptor* knockout (*Ldlr−/−*) mice, female mice fed normal chow or atherosclerotic diet for less than 6 months generally have a greater atherosclerotic lesion size and overall burden than their male counterparts. In our study, the *Apoe−/−* mice were euthanized at less than 6 months of age (20 weeks), and the female atherosclerotic plaques are expected to be larger than the male atherosclerotic plaques at this age, which may have affected our experimental results. Considering the possible impact of sex differences in experimental animals on the results and the epidemiological differences in the incidence of atherosclerosis in males, our study only included male mice.

We have thought about addressing the problem of including males and females in future experiments. One possibility is to increase the sample size and group the mice (both male and female) randomly. The random grouping method should work but will require a sufficiently large sample size. If the sample size is too small, the influence of sex differences will impact the ability to obtain statistically significant findings. The second possibility is to group the female mice separately, and then compare the differences between males and females. However, the focus of such a study will be biased towards sex comparisons rather than mechanistic research. Regardless of the chosen method, studies involving both males and females are beyond the resource limitations of the current study and are planned for future experimentation.

2) The authors mentioned that they have included picture of CTSB protein expression in mouse aorta at 20 weeks of age in figure 2C, but I was not able to locate those figures.

Response: We are very sorry for our negligence regarding the picture that we mentioned, which was not included in the manuscript and reply letter. Due to figure space limitations, we intended to put the picture in the reply letter and provide the western blot quantification of CTSB in Figure 2C. However, because of issues displaying the image in the editorial system, we placed the picture in Figure 1A but forgot to change our response to the reviewer accordingly. We are sorry for the inconvenience this has caused. The plotted results in Figure 2C show that CTSB expression persisted at 20 weeks. The results in Figure 1A show that plaques appeared in the aorta of virus-transduced mice fed high-fat diet, and EGFP fluorescence was present in the plaques. For convenience, Figure 1 is also shown below.

3) I could not locate the alpha-actin SMC staining of the cultured SMCs that the author mentioned in their response. Also, in their response to my comment #9, they refer to their results at the end, but I couldn't locate those either.

Response: We are sorry about the picture that was missing from the revised manuscript. We believe that the problem occurred when uploading files to the editorial system. We re-examined these images and added them to Figure 1I and 1J to ensure that they can be found by the reviewer. The results are as follows:

We tried our best to improve the manuscript by addressing reviewer concerns. These changes did not influence the content and framework of the paper. We appreciate the helpful and earnest critiques of the Editors and reviewers and hope that the revisions will meet with their approval.

We would like to thank you for allowing us to resubmit a revised copy of the manuscript and we highly appreciate your time and consideration. Please do not hesitate to contact us if there are any questions. Thank you again for your hard work! Best wishes to you!

Sincerely.

Danan Liu.
